# Numerical Simulation Study on the Influence of MWCNT and Genipin Crosslinking on the Actuation Performance of Artificial Muscles

**DOI:** 10.3390/biomimetics11010028

**Published:** 2026-01-02

**Authors:** Zhen Li, Yunqing Gu, Chendong He, Denghao Wu, Zhenxing Wu, Jiegang Mou, Caihua Zhou, Chengqi Mou

**Affiliations:** 1 College of Metrology Measurement and Instrument, China Jiliang University, Hangzhou 310018, China; p24020854063@cjlu.edu.cn (Z.L.); p20020854018@cjlu.edu.cn (C.H.); wdh@cjlu.edu.cn (D.W.); wzx2022@cjlu.edu.cn (Z.W.); mjg@cjlu.edu.cn (J.M.); 12127107@zju.edu.cn (C.M.); 2 Depamu (Hangzhou) Pumps Technology Co., Ltd., Hangzhou 310018, China; zhoucaihua@depamu.com; 3 College of Energy Engineering, ZheJiang University, Hangzhou 310027, China

**Keywords:** artificial muscle, MWCNT, Genipin, actuation performance, bending force, displacement

## Abstract

To enhance the actuation performance of artificial muscles, a thermo-piezoelectric coupled model was developed based on the inverse piezoelectric effect of piezoelectric bimorphs. By altering the effective piezoelectric coefficient, elastic modulus, and effective thermal expansion coefficient of the thermo-piezoelectric bimorph model, the bending motion of artificial muscles was simulated. The effects of multi-walled carbon nanotube (MWCNT) and Genipin crosslinking on the bending force and output displacement of the artificial muscles were analyzed, illustrating how crosslinking affects the equivalent actuation response. The results showed that MWCNT and Genipin crosslinking significantly improved the actuation performance of the artificial muscles. Through numerical simulation, the optimal crosslinking ratio was determined to be 43.34% MWCNT and 0.1% Genipin, at which the best actuation performance was achieved. Compared to non-crosslinked techniques, the artificial muscles with crosslinked structures exhibited markedly enhanced actuation behavior.

## 1. Introduction

With the rapid advancement in the fields of biological and intelligent materials, artificial muscles—intelligent materials designed to simulate the functions of biological muscles—have attracted extensive research attention [1]. The development of novel artificial muscle systems has thus become increasingly critical, as they demonstrate tremendous application potential in domains such as medical rehabilitation, robotic motion control, and aerospace engineering [2,3,4,5,6,7]. Drawing inspiration from the mechanisms of natural muscles, researchers have developed diverse types of artificial muscles, including film-based [8] and fiber-based artificial muscles [9]. As a natural biopolymer [10], chitosan, often processed into forms such as films, fibers, or hydrogels [11], is widely employed in the fabrication of artificial muscles owing to its superior biodegradability [12] and film-forming properties [13]. In particular, chitosan hydrogel, due to its biocompatibility, ionic conductivity, and tunable mechanical properties [14], has emerged as a promising material for constructing soft, responsive artificial muscle systems. However, existing artificial muscle technologies still face several limitations, including low energy efficiency, slow response, and limited durability. Among the key metrics for evaluating the actuation performance of artificial muscles, driving force [15] and response speed [16] are of particular importance. However, the intricate internal mechanisms of chitosan-based artificial muscles, coupled with the relative scarcity of relevant research, have impeded their further development and practical application. Therefore, exploring and optimizing the preparation processes of artificial muscles is of significant importance for enhancing their overall performance.

For artificial muscles fabricated via the direct casting and assembly process, the tight adhesion between the electroactive membrane and non-metallic electrode membrane results in low contact resistance, which significantly enhances the electrical conductivity of the muscles and nearly doubles their response speed [17]. Heat treatment technology also influences the response speed of the muscles: as the heat treatment duration increases, the response speed of the muscles improves continuously; however, with prolonged heating, the electromechanical performance of the artificial muscles begins to deteriorate [18]. In the fabrication of artificial muscles, biological crosslinking techniques are commonly employed [19]. When artificial muscles are crosslinked with Genipin at varying concentrations, although uncrosslinked artificial muscles exhibit a faster initial reaction rate, over time, Genipin-crosslinked artificial muscles demonstrate increasingly superior stability, thereby displaying significant advantages. With the advancement of scientific research, novel crosslinking methods have been applied, such as the preparation of a Genipin-chitosan crosslinked actuation layer and a multi-walled carbon nanotube (MWCNT)-graphene crosslinked electrode layer. Due to the unique properties of MWCNT, Researchers have attempted to integrate them together. Actuation characteristics can be effectively optimized by adjusting the addition ratios of MWCNT and Genipin [20]. MWCNT, endowed with excellent electrical, mechanical, and thermal properties [21,22], shows broad application prospects in nanocomposites [23,24], energy sectors, and cutting-edge scientific fields [25,26]. Genipin, with its excellent biocompatibility [27] and efficient crosslinking ability [28,29,30], has been widely applied in tissue engineering [31] and the preparation of biomaterials [32]. Researchers have found that the incorporation of Genipin and MWCNT plays a crucial role in enhancing the water retention capacity of bionic artificial muscles, significantly improving their durability, while also exerting an influence on their actuation performance [33].

In summary, most studies on artificial muscle actuation are experimental, requiring substantial material and equipment use. Numerical simulation serves as a valuable complementary tool to enhance efficiency and reduce trial complexity. Therefore, in this study, a piezoelectric bimorph model was established, and an equivalent thermal-piezoelectric coupled bimorph model was optimized by means of coupled analysis of thermal field and piezoelectric model. The equivalent thermal-piezoelectric coupled bimorph model was used to simulate the actuation characteristics of bionic artificial muscles under different working conditions, and response surface optimization experiments were carried out for three factors, namely the addition ratio of MWCNT, the addition ratio of Genipin and the driving voltage. Finally, the optimal addition ratios of MWCNT, Genipin and voltage were determined. This method reduces the number of repeated physical experiments by enabling rapid evaluation of parameter effects in simulation, rather than replacing experimental procedures, and promotes the development of bionic artificial muscles. Compared with previous studies that rely primarily on experimental characterization, this work introduces a equivalent thermo-piezoelectric numerical modeling framework that enables systematic evaluation of the effects of MWCNT and Genipin crosslinking, representing a methodological innovation for chitosan-based artificial muscle research.

## 2. Modeling and Numerical Simulation Analysis

### 2.1. Establish a Thermo-Piezoelectric Model of Artificial Muscles

When voltages with opposite directions are applied to the positive and negative electrodes of a piezoelectric wafer, the resulting electric field may align with or oppose the polarization direction, causing the wafer to elongate or contract accordingly. Consequently, the piezoelectric bimorph bends under the combined actuation of the two wafers. The actuation mechanism of the biomimetic artificial muscle is analogous to that of the piezoelectric bimorph; therefore, the bimorph model can be used to simulate the actuation behavior of the artificial muscle. Compared with ion-polymer actuator models, the present approach offers a simpler and more computationally efficient way to capture bending behavior through equivalent parameters. However, the deformation of the artificial muscle is inherently nonlinear, while that of the piezoelectric bimorph follows a linear relationship. Directly substituting the parameters of the artificial muscle into the bimorph model would thus introduce significant errors. Considering that temperature variations often accompany the electrical stimulation of artificial muscles, a thermo-piezoelectric coupling analysis is required. Hence, the conventional piezoelectric bimorph model was extended to a thermo-piezoelectric coupled bimorph model to enhance simulation accuracy and physical consistency. Based on the finite element method (FEM), the equivalent thermo-piezoelectric coupling module (Thermal-Piezoelectric Body) in ANSYS Workbench 2022 R2 was employed to simulate the biomimetic artificial muscle. The simulation involved defining the material properties of the artificial muscle and determining the equivalent piezoelectric coefficients, elastic modulus, and thermal expansion coefficients required for the coupled analysis. The relationships between the equivalent piezoelectric coefficients and elastic modulus were derived from the tip displacement and tip force equations of the bimorph model.

**Figure 1:**
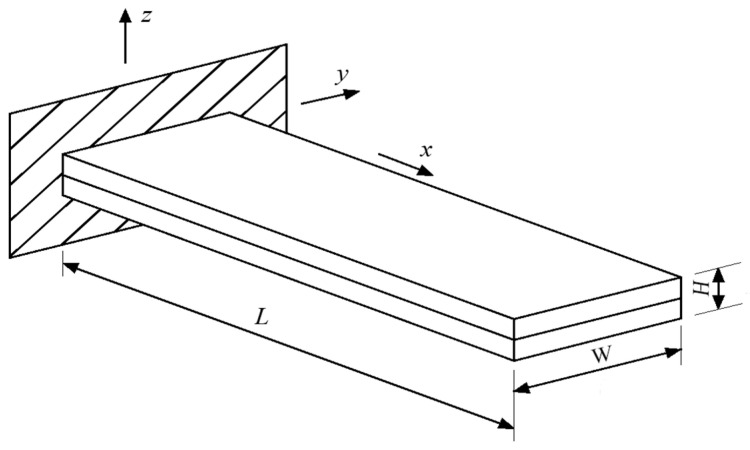
Piezoelectric bimorph model of the bionic artificial muscle.

The piezoelectric bimorph model of the bionic artificial muscle is shown in Figure 1, and its tip displacement *S* and tip force *F*_b_ are given by Equations (1) and (2).

(1) $$S=\frac{3L^{2}(1+b)(2b+1)}{2H(ab^{3}+3b^{2}+3b+1)}d_{31}E_{3}$$

(2) $$F_{b}=\frac{3WH^{2}E}{8L}\frac{2b+1}{{\left(b+1\right)}^{2}}d_{31}E_{3}$$

In the equations, *L* is the length of the piezoelectric model (mm), *H* is the thickness of the piezoelectric model (mm), *W* is the width of the piezoelectric model (mm), *a* is the ratio of the elastic modulus of the middle elastic layer to that of the outer layer, *b* is the ratio of the thickness of the middle elastic layer to that of the outer layer, *d*_31_ is the equivalent piezoelectric coefficient (mm/V), *E*_3_ is the electric field intensity (V/mm), *E* is the elastic modulus (Pa), *S* is the tip displacement of the piezoelectric model (mm), *F*_b_ is the tip force of the piezoelectric model (mN).

Since the piezoelectric bimorph model undergoes continuous deformation and the middle driving layer is relatively thin, the elastic layer can be neglected, and thus *a* = *b* = 0. As *V* = *E*_3_*H*, the formulas for the equivalent piezoelectric coefficient and elastic modulus can be derived from Equations (1) and (2), where *V* denotes the voltage applied to the piezoelectric model (V).

(3) $$d_{31}=\frac{2SH^{2}}{3L^{2}V}$$

(4) $$E=\frac{8LF_{b}}{3WHd_{31}V}$$

The equivalent thermal expansion coefficient is then derived from the equivalent piezoelectric coefficient, as expressed below:

(5) $$a=\frac{d_{31}}{M}\frac{\Delta M\;}{\;\Delta T}$$

In the equations: α is the equivalent thermal expansion coefficient (1/°C), M is the thickness of a single piezoelectric layer, M = H/2 (mm), ΔM is the change in the thickness of a single piezoelectric layer (mm) and ΔT is the change in temperature (°C). By defining the material properties of the artificial muscle as well as the equivalent piezoelectric coefficients, elastic moduli, and equivalent thermal expansion coefficients required for the simulation of the thermo-piezoelectric module, the relational expressions between the equivalent piezoelectric coefficients and elastic moduli were derived using the tip displacement and tip force equations of the piezoelectric bimorph model. Experimental data of bionic artificial muscles with different addition ratios of MWCNT and Genipin were selected [20], and the equivalent piezoelectric coefficients, elastic moduli, and equivalent thermal expansion coefficients of their respective thermo-piezoelectric bimorph models were calculated, as shown in Table 1. Subsequently, each parameter was substituted into the thermo-piezoelectric coupling module of ANSYS for simulation. The addition ratios of MWCNT are 0% (0 mL), 40% (4 mL), 60% (6 mL), while the addition ratios of Genipin are 0% (0 mg), 0.1% (0.5 mg), 0.2% (1 mg).

**Figure 2:**
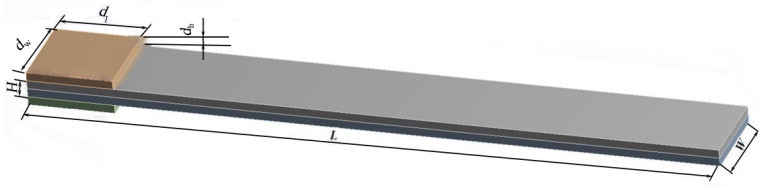
Physical model of bionic artificial muscle.

Using the thermo-piezoelectric bimorph model developed above, we established a finite-element framework to simulate the system. The physical model of the artificial muscle was established using SolidWorks 2022, as shown in Figure 2. Copper sheets are fixed on the upper and lower sides of the model, serving as the driving power source for energizing the artificial muscle in the numerical simulation; the middle two layers are the piezoelectric bimorph model of the artificial muscle. *L* = 40 mm, *W* = 5 mm, *H* = 1 mm, *d*_l_ = 5 mm, *d*_w_ = 5 mm, *d*_h_ = 0.5 mm.

### 2.2. Meshing of the Model

The model was imported into ANSYS Workbench for meshing. Due to the relatively simple structure of the overall model, structured hexagonal meshes were adopted to improve accuracy. Mesh independence verification was conducted, with mesh sizes of 0.05 mm, 0.075 mm, 0.1 mm, 0.25 mm, and 0.5 mm respectively. The simulation results for these five different mesh sizes are shown in Table 2.

**Figure 3:**
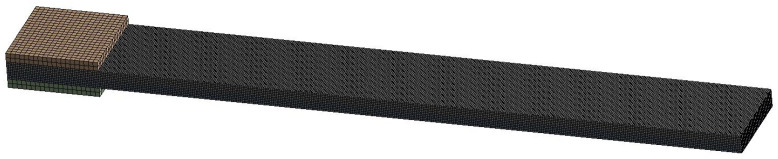
Mesh division of the artificial muscle simulation mode.

By comparing with the simulation results of the finest mesh, it can be found that the relative errors of the simulation results for the four groups of different mesh sizes are all within 0.5%, which indicates that the mesh size has a very small impact on the simulation calculation of this model. Among them, the mesh size of 0.1 mm has high calculation accuracy and a reasonable total number of meshes, so the mesh size of 0.1 mm was selected for dividing the simulation model. Since the simulation analysis mainly focuses on the characteristic changes in the middle piezoelectric bimorph, the mesh size of the copper sheet electrodes on both sides was set to 0.25 mm to reduce the demand for computing resources, with the total number of meshes being 201,600. The meshing of the model is shown in Figure 3.

The bending behavior of the cantilever beam mimics the bending and contraction of muscle fibers. The fixed-end configuration not only reduces complex boundary constraints but also facilitates the estimation of the driving force, displacement range, and response time of the artificial muscle. Therefore, a cantilever-beam structure was adopted in the simulation. The end surface with copper electrodes was fixed to represent the clamped end of the biomimetic artificial muscle. Subsequently, voltage loads are applied to the copper sheet electrodes on both sides of the model, with 5 V set on one side and 0 V on the other, so as to simulate the energized state of the bionic artificial muscle. According to the equivalent piezoelectric coefficient, elastic modulus, and equivalent thermal expansion coefficient of the unoptimized artificial muscle listed in Table 1, the material properties and main parameters of the equivalent thermo-piezoelectric coupling module were set as shown in Table 3. After completing the parameter settings, simulations of the output displacement and output force were carried out. It should be noted that the simplified linear equivalent-bimorph formulation is most accurate under moderate deformation and low-to-medium loading conditions. Under high load or large strain, deviations may increase, and this limitation has been explicitly acknowledged.

## 3. Analysis of the Characteristics of Artificial Muscles Under Different Addition Ratios

### 3.1. The Influence of Different Addition Ratios of MWCNT and Genipin on Actuation Performance

**Figure 4:**
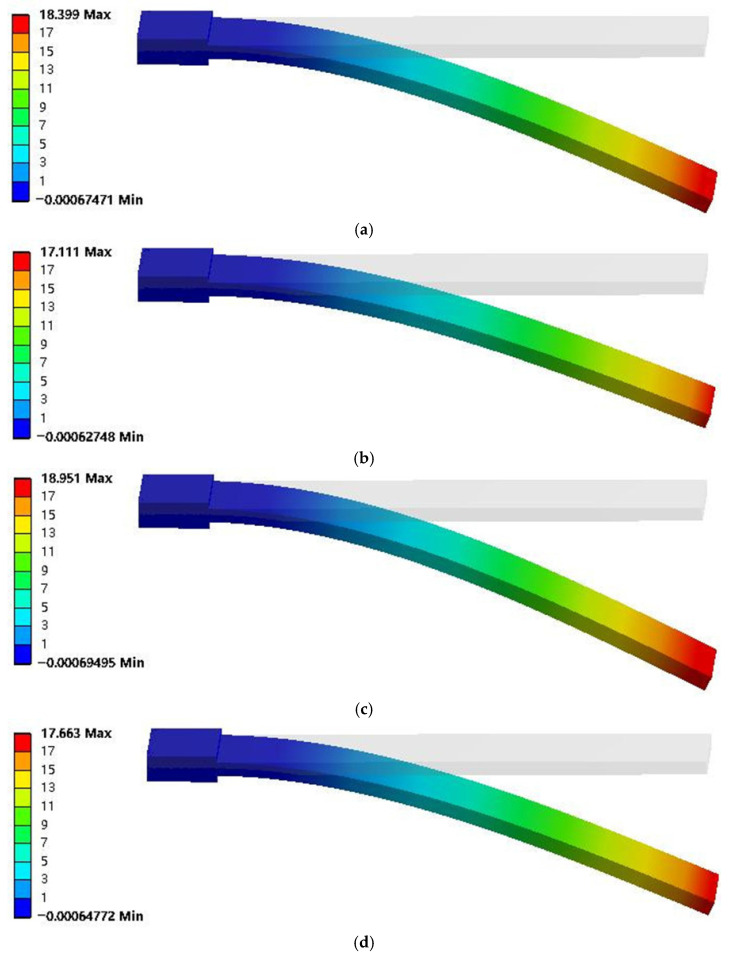
Displacement deformation contour diagrams of simulation models for different artificial muscles, (**a**) 440. MWCNT, 0.1%Genipin, (**b**) 40%MWCNT, 0.2%Genipin, (**c**) 60%MWCNT, 0.1%Genipin, (**d**) 60%MWCNT, 0.2%Genipin.

**Figure 5:**
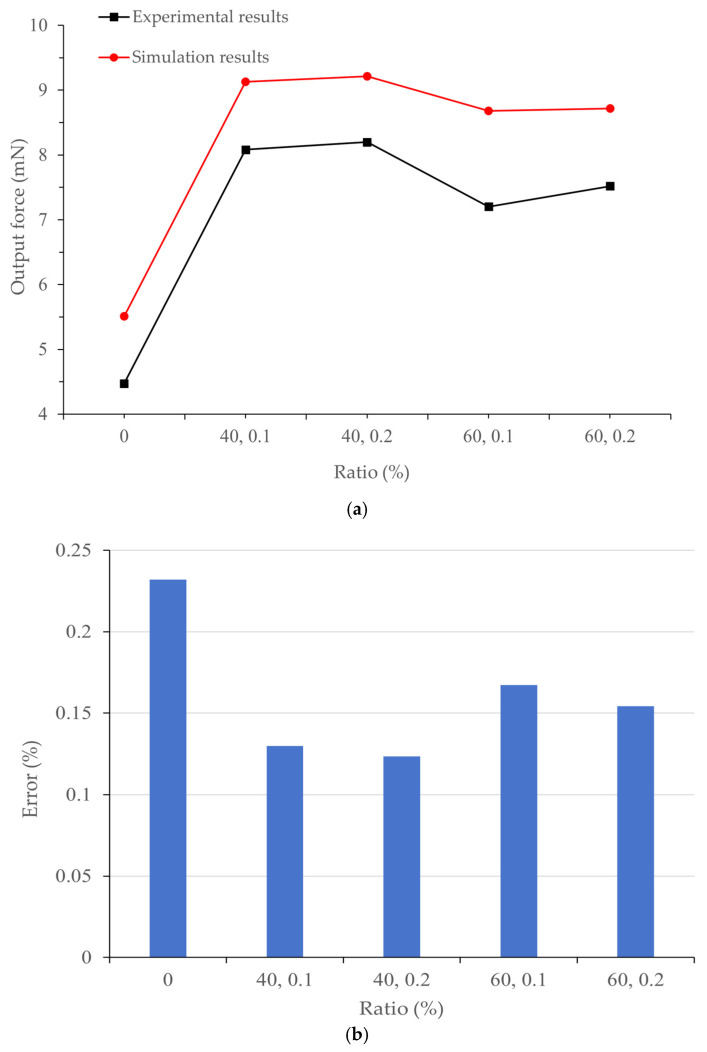
(**a**) Comparison between the simulation results and experimental results [20] of the output force of different artificial muscles; (**b**) Prediction error between simulation and experimental results.

The parameters of artificial muscles with different MWCNT and Genipin addition ratios in Table 1 were substituted into the model to simulate the deflection displacement (mm) and bending force (mN). The simulation displacement deformation nephogram is shown in Figure 4, and the output force results of the simulation model are shown in Figure 5.

It can be seen from Figure 4 that the artificial muscle has the smallest displacement deformation under crosslinking with 40% MWCNT and 0.2% Genipin, which is 17.111 mm; while it has the largest displacement change under crosslinking with 60% MWCNT and 0.1% Genipin, which is 18.951 mm. By comparing with the experimental images captured by a high-speed camera, it is found that the displacement change pattern of the simulation model is consistent with the actual displacement change pattern of the artificial muscle. Moreover, in both cases, the artificial muscle with the addition ratio of 60% MWCNT and 0.1% Genipin has the largest output displacement, which is consistent with the experimental results. It can be seen from Figure 5 that the output force of the thermo-piezoelectric bimorph model is also similar to the experimental data in terms of magnitude and variation trend. In addition, the improvement multiple of the maximum displacement deformation of the crosslinked artificial muscle is approximately the same as that of the output displacement of the artificial muscle compared with the non-crosslinked one in the respective experiments [20]. This indicates that the thermo-piezoelectric coupled bimorph model can be used to simulate the movement and deflection process of bionic artificial muscles.

To further analyze the calculation accuracy of the simulation model, an error analysis was performed on the simulation data of artificial muscles with different addition ratios. The simulation displacement data at a position 30 mm away from the end face of the copper sheet electrode were read. A comparative verification of the simulation data and experimental data regarding the output displacement and output force of different artificial muscles is shown in Table 4.

As can be seen from Table 4, although the maximum errors of the output displacement and output force of the bionic artificial muscle without crosslinking agent are 20.11% and 23.2% respectively, the simulation errors of the artificial muscles optimized by additive crosslinking are kept in the range of about 10% to 15%, and the errors are continuously decreasing with the increase in the experimental output force and displacement of the bionic artificial muscles. This indicates that with the improvement of the output performance of the artificial muscle, its change trend is more in line with the regularity of the simulation model, verifying the reliability of the thermo-piezoelectric coupled bimorph model for bionic artificial muscles. Numerical simulation has proved that the thermo-piezoelectric coupled bimorph model conforms to the actuation mechanism of artificial muscles. Compared with traditional experimental methods, using this model to analyze the mechanical response characteristics of artificial muscles under different environments and study the impact of different addition ratios of MWCNT and Genipin on the actuation performance of artificial muscles not only saves costs but also has better visualization effects, which can better reflect the changes in displacement and output force after changing the proportion.

### 3.2. Analysis of the Actuation Characteristics of Artificial Muscles Under Different Voltages

**Figure 6:**
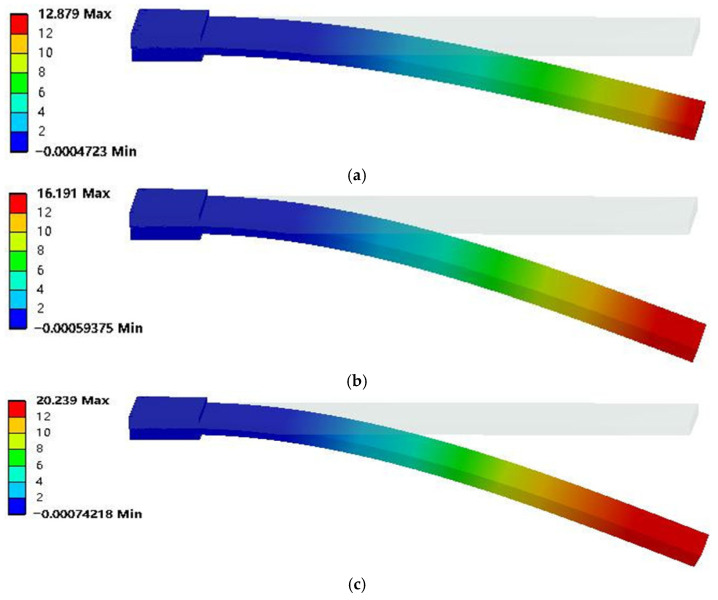
Displacement deformation contour diagrams of artificial muscles under different voltages, (**a**) 1 V, (**b**) 4 V, (**c**) 7 V.

The suitable energizing voltage for the bionic artificial muscle ranges from 1 V to 7 V. Moreover, since the response surface methodology with three factors and three levels will be subsequently used to optimize the bionic artificial muscle, this model was employed to simulate the actuation characteristics under the minimum voltage of 1 V, the maximum voltage of 7 V, and the intermediate voltage of 4 V. The artificial muscle with the addition ratio of 60% MWCNT and 0.3% Genipin was simulated under different voltages, and the displacement deformation nephogram is shown in Figure 6.

It can be seen from Figure 6 that the simulated displacement at the tip of the artificial muscle is 12.879 mm, 16.191 mm, and 20.239 mm under voltages of 1 V, 4 V, and 7 V, respectively. As the voltage increases, the simulated displacement at the tip of the artificial muscle gradually increases with an obvious linear growth trend. On average, the displacement increases by 0.9 mm for every 1 V increase in voltage. The above research shows that the deflection displacement of the bionic artificial muscle can also be significantly improved by increasing the driving voltage, and the improvement effect is optimal when the driving voltage is 7 V, which is 1.57 times that of the control group. The simulation data of the actuation characteristics of the artificial muscle under different MWCNT and Genipin addition ratios as well as different driving voltages were recorded, and will be optimized using the response surface methodology subsequently.

## 4. Response Surface Optimization of Bionic Artificial Muscles

### 4.1. Experimental Design of Response Surface Methodology

In this experiment, the addition ratio of MWCNT, the addition ratio of Genipin, and the driving voltage were selected as factor variables, with deflection displacement and bending force as response values. Based on the Box–Behnken Design (BBD) module in Design-Expert (Version 13), an experimental design for process parameter optimization with three factors and three levels was conducted. Considering that the bionic artificial muscles exhibit better actuation performance when the MWCNT addition ratio ranges from 20% to 60% and the Genipin addition ratio ranges from 0.1% to 0.3%, the above ratios were adopted as the level values for the corresponding factors. The experimental factors and their level values for the response surface are shown in Table 5.

According to the design principle of BBD, the response surface experiment was designed, resulting in 17 sets of experimental conditions. Simulations were conducted on the deflection displacement and bending force under the corresponding conditions, and the results are shown in Table 6.

By fitting the experimental data in Table 6, polynomial regression equation models for the deflection displacement *S*x and bending force *F*x, which are fitted by three factors including MWCNT addition ratio (A), Genipin addition ratio (B), and driving voltage (C), were obtained respectively, as shown in Formulas (6) and (7).

(6) $$\begin{array}{l} S_{x}=15.98+0.36A-0.56B+3.09C-0.0055AB+0.048AC-0.11BC\\ -0.2A^{2}+0.22B^{2}-0.048C^{2}+0.32A^{2}B-0.39A^{2}C-0.018AB^{2} \end{array}$$

(7) $$\begin{array}{l} F_{b}=8.31+0.058A-0.032B+2.67C-0.11AB-0.15AC+0.016BC\\ -0.47A^{2}-0.31B^{2}+0.33C^{2}+0.034A^{2}B+0.19A^{2}C+0.039AB^{2} \end{array}$$

In the equations: *S*_x_ is the deflection displacement (mm); *F*x is the bending force (mN); *A* is the MWCNT addition ratio (%); *B* is the Genipin addition ratio (%); and *C* is the driving voltage (V).

The analysis of variance (ANOVA) results corresponding to the deflection displacement *S_x_* and bending force *F_x_* are shown in Table 7 and Table 8, respectively. The significance of the fitting of the regression equation models was evaluated by analyzing the variance results. When the *p*-value < 0.0001, it indicates that the response value of the corresponding variable is highly significant; the larger the mean square, the higher the degree of influence of the corresponding variable. It can be observed that the *p*-values of both regression equation models are less than 0.001, indicating that the adopted fitting regression models are highly significant. According to the magnitude of the mean square values, the three factors affecting the deflection displacement are in the order of voltage (*C*), Genipin addition ratio (*B*), and MWCNT addition ratio (*A*); while the three factors affecting the bending force are in the order of voltage (*C*), MWCNT addition ratio (*A*), and Genipin addition ratio (*B*). This shows that voltage is the main factor affecting the actuation characteristics of the bionic artificial muscle. The correlation coefficients *R*^2^ for the fitting degrees of the regression equation models for deflection displacement and bending force are 0.9927 and 0.9988, respectively, indicating that they can explain 99.27% and 99.88% of the response values, respectively, with good correlation.

### 4.2. Results and Interpretation

**Figure 7:**
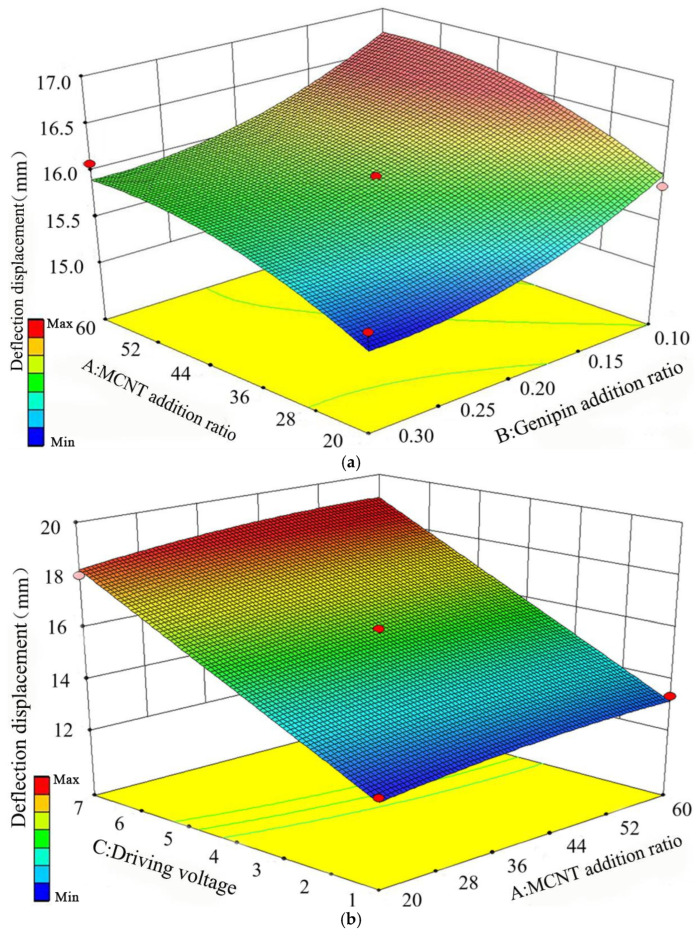

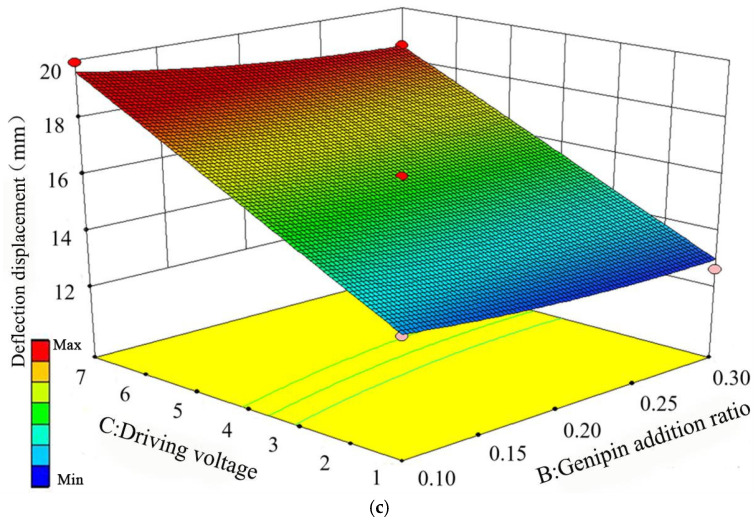
Response results of pairwise interactions of the three factors on deflection displacement, (**a**) Addition ratios of MWCNT and Genipin, (**b**) MWCNT addition ratio and driving voltage, (**c**) Genipin addition ratio and driving voltage.

By performing regression fitting on the data in Table 6, three-dimensional response surfaces reflecting the effects of pairwise interactions among the three factors on the deflection displacement *S*x and bending force *F*x were obtained. Through the analysis of these response surfaces, the degree of influence of each variable on *S*x and *F*x, as well as the optimal process parameters, can be derived. The response results of the pairwise interactions among the three factors on the deflection displacement are shown in Figure 7.

It can be seen from Figure 7a that when the MWCNT addition ratio is fixed, as the Genipin addition ratio gradually decreases within the range of 0.3% to 0.1%, the deflection displacement continues to increase, and the increasing rate is constantly accelerating. When the Genipin addition ratio is fixed, as the MWCNT addition ratio gradually increases within 20% to 60%, the deflection displacement also keeps rising, but the increasing rate is decreasing and gradually slowing down. The increasing rate of Genipin addition ratio on deflection displacement shows a semi-parabolic shape, and its curvature is slightly larger than that of MWCNT addition ratio, indicating that the Genipin addition ratio has a more significant impact on deflection displacement compared with the MWCNT addition ratio.

It can be seen from Figure 7b that when the driving voltage is fixed, the deflection displacement increases continuously as the MWCNT addition ratio gradually increases within 20% to 60%. When the MWCNT addition ratio is fixed, the deflection displacement keeps increasing with the gradual increase in driving voltage within 1 V to 7 V. The increasing rate of driving voltage on deflection displacement is much greater than that of MWCNT addition ratio, showing a linear growth trend.

**Figure 8:**
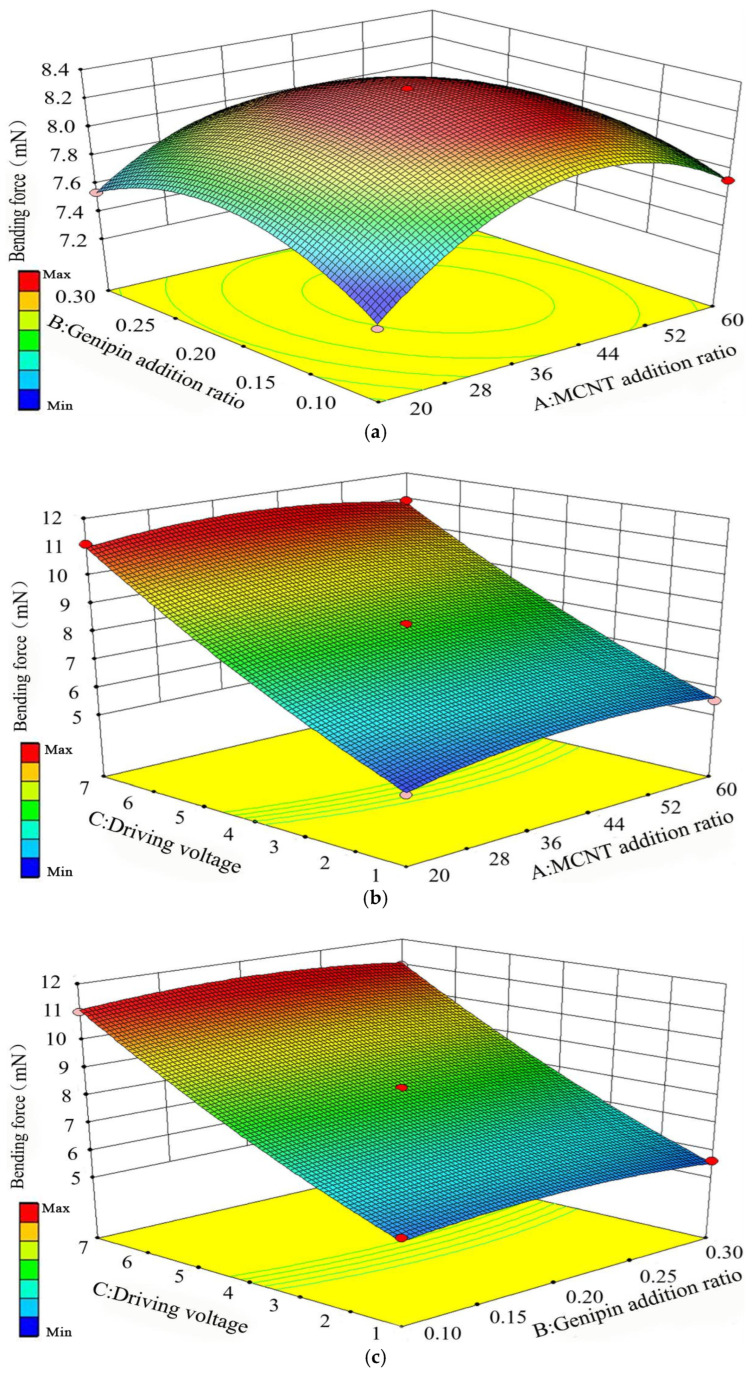
Response results of pairwise interactions among the three factors on bending force, (**a**) Addition ratios of MWCNT and Genipin, (**b**) MWCNT addition ratio and driving voltage, (**c**) Genipin addition ratio and driving voltage.

It can be seen from Figure 7c that when the Genipin addition ratio is fixed, the deflection displacement increases continuously as the driving voltage gradually increases within 1 V to 7 V. When the driving voltage is fixed, the deflection displacement keeps increasing with the gradual decrease in Genipin addition ratio within 0.3% to 0.1%. The increasing rate of driving voltage on deflection displacement is much greater than that of Genipin addition ratio. To sum up, the driving voltage is the main factor affecting the deflection displacement, followed by the Genipin addition ratio and the MWCNT addition ratio in sequence. The optimal process parameters for improving the deflection displacement are 45.3% MWCNT, 0.1% Genipin, and a driving voltage of 7 V. The response results of the pairwise interactions among the three factors on the bending force are shown in Figure 8.

It can be seen from Figure 8a that when the MWCNT addition ratio is fixed, the bending force shows a trend of first increasing and then decreasing regardless of whether the Genipin addition ratio increases or decreases. When the Genipin addition ratio is fixed, the bending force also shows a trend of first increasing and then decreasing regardless of whether the MWCNT addition ratio increases or decreases, which indicates that the interaction between the two factors is significant. The increasing rate of the MWCNT addition ratio on the bending force presents a parabolic shape, and its curvature is slightly larger than that of the Genipin addition ratio, suggesting that the MWCNT addition ratio has a more significant impact on the bending force compared with the Genipin addition ratio.

It can be seen from Figure 8b that when the driving voltage is fixed, the bending force shows a trend of first increasing and then decreasing regardless of whether the MWCNT addition ratio increases or decreases. When the MWCNT addition ratio is fixed, the bending force increases continuously as the driving voltage gradually increases within the range of 1 V to 7 V. The increasing rate of the driving voltage on the bending force is much greater than that of the MWCNT addition ratio, which is the same as the case of deflection displacement, and it shows a linear growth trend.

It can be seen from Figure 8c that when the Genipin addition ratio is fixed, the bending force increases continuously as the driving voltage gradually increases within 1 V to 7 V. When the driving voltage is fixed, the bending force shows a trend of first increasing and then decreasing regardless of whether the Genipin addition ratio increases or decreases. The increasing rate of the driving voltage on the bending force is much greater than that of the Genipin addition ratio. To sum up, the driving voltage is the main factor affecting the bending force, followed by the MWCNT addition ratio and the Genipin addition ratio in sequence. The optimal process parameters for improving the bending force are 36.8% MWCNT, 0.18% Genipin, and a driving voltage of 7 V.

The multi-response optimization was performed using a standard desirability function with equal weights assigned to displacement and bending force. A qualitative sensitivity analysis showed that ±5% deviations in the input ratios lead to 8% variation in the predicted responses. The optimal parameter combination was determined by applying a multi-response desirability optimization to Equations (6) and (7), where displacement and force were simultaneously maximized. For the polynomial regression equation models of deflection displacement and bending force, with the goal of maximizing the combination of deflection displacement and bending force, the optimal process parameters calculated are 43.34% MWCNT addition ratio, 0.1% Genipin addition ratio, and 7 V driving voltage, with a deflection displacement of 19.987 mm and a bending force of 11.033 mN. The simulation verification using the optimal process parameters to test the reliability of the regression equation shows that the deflection displacement is 20.398 mm and the bending force is 11.347 mN. The errors of the deflection displacement and bending force in the simulation are 2.01% and 2.77% respectively, which are within the normal range. The regression equation model has high reliability and a significant optimization effect on the actuation performance of chitosan gel artificial muscles.

## 5. Conclusions

The model established in this study can be effectively used for the numerical simulation analysis of the actuation performance of artificial muscles with varying MWCNT and Genipin addition ratios, demonstrating promising application prospects. The proposed equivalent thermos-piezoelectric framework can be extended to other polymer-based bending actuators as long as their effective electromechanical coefficients can be experimentally calibrated. However, its accuracy may decrease for actuators dominated by ion migration or poroelastic processes, which should be considered in future work.

(1) When the MWCNT addition ratio ranges from 20% to 60% and the Genipin addition ratio ranges from 0.1% to 0.3%, the increase in both addition ratios leads to a trend where the output force of the artificial muscle first increases and then decreases. The displacement of the artificial muscle is positively correlated with the MWCNT addition ratio, but the increasing rate keeps decreasing. In contrast, the displacement is negatively correlated with the Genipin addition ratio: as the Genipin addition ratio decreases, the deflection displacement continues to increase, and the increasing rate is constantly accelerating.
(2) Increasing the driving voltage can also significantly enhance the deflection displacement of the bionic artificial muscle. As the voltage increases, the simulated displacement at the tip of the artificial muscle shows a linear growth trend, with an average increase of 0.9 mm in displacement for every 1 V increase in voltage.
(3) Under the conditions of 43.34% MWCNT addition ratio, 0.1% Genipin addition ratio, and a driving voltage of 7 V, the chitosan gel artificial muscle exhibits the optimal actuation performance, with a deflection displacement of 20.398 mm and a bending force of 11.347 mN.

## Figures and Tables

**Table 1:** Parameters of the piezoelectric model for artificial muscles with different MWCNT and Genipin addition ratios.

MWCNT Addition Ratio	Genipin Addition Ratio	Equivalent Piezoelectric Coefficient (10^−3^ mm/v)	Elastic Modulus (mpa)	Equivalent Thermal Expansion Coefficient (10^−3^/°C)
0% (0 mL)	0% (0 mg)	0.315	0.606	0.945
40% (4 mL)	0.1% (0.5 mg)	0.823	0.419	2.47
40% (4 mL)	0.2% (1 mg)	0.728	0.480	2.185
60% (6 mL)	0.1% (0.5 mg)	0.841	0.365	2.523
60% (6 mL)	0.2% (1 mg)	0.773	0.415	2.32

**Table 2:** Simulation results of four different mesh sizes.

Mesh Size (mm)	Total Number of Meshes	Displacement Deformation (mm)	Relative Error
0.5	1800	7.7099	0.426
0.25	14,400	7.7271	0.204
0.1	225,000	7.7366	0.081
0.075	563,738	7.7411	0.232
0.05	1,800,000	7.7429	—

**Table 3:** Simulation Parameter Settings.

Property	Value	Unit
Density	2750	kg/m^3^
Equivalent thermal expansion coefficient	9.75 × 10^−4^	1/°C
Equivalent piezoelectric coefficient	3.15 × 10^−4^	mm/V
Elastic modulus	0.606	MPa
Poisson’s ratio	0.45	—
Bulk modulus	2.02 × 10^6^	Pa
Shear modulus	2.09 × 10^5^	Pa
*e*_31_	4_11_	C/m^2^
*e*_33_	6100	C/m^2^
*e*_15_	0	C/m^2^
*ep*_11_	0.5	—
*ep*_33_	0.5	—

**Table 4:** Experimental factors and their level values.

MWCNT Addition Ratio (%)	Genipin Addition Ratio (%)	Displacement (mm)			Output Force (mN)		
—	—	Simulation	Experiment [20]	Error (%)	Simulation	Experiment [20]	Error (%)
0 (0 mL)	0 (0 mg)	4.54	3.78	20.11	5.512	4.474	23.2
40 (40 mL)	0.1 (0.5 mg)	11.01	9.88	11.44	9.131	8.081	12.99
40 (40 mL)	0.2 (1 mg)	10.08	8.74	15.33	9.213	8.199	12.36
60 (60 mL)	0.1 (0.5 mg)	11.15	10.09	10.51	8.679	7.202	16.73
60 (60 mL)	0.2 (1 mg)	10.51	9.28	13.25	8.716	7.519	15.42

**Table 5:** Experimental factors and their levels.

Experimental Factor	Code	Level Value		
MWCNT addition ratio (%)	*A*	20	40	60
Genipin addition ratio (%)	*B*	0.1	0.2	0.3
Driving voltage (V)	*C*	1	4	7

**Table 6:** Response surface experimental design and results.

Code	MWCNT Addition Ratio (%)	Genipin Addition Ratio (%)	Driving Voltage (V)	Deflection Displacement (mm)	Bending Force (mN)
1	60	0.2	7	18.836	10.936
2	60	0.1	4	16.593	7.732
3	60	0.2	1	13.348	5.523
4	20	0.1	4	15.891	7.316
5	40	0.1	1	13.511	5.712
6	40	0.3	1	12.615	5.616
7	40	0.2	4	15.981	8.312
8	40	0.2	4	15.981	8.312
9	60	0.3	4	16.091	7.513
10	20	0.2	7	18.012	11.112
11	20	0.2	1	12.718	5.114
12	40	0.2	4	15.981	8.312
13	40	0.1	7	19.917	11.01
14	40	0.2	4	15.981	8.312
15	20	0.3	4	15.411	7.542
16	40	0.3	7	18.566	10.978
17	40	0.2	4	15.981	8.312

**Table 7:** Analysis of variance for the regression equation of deflection displacement *S*x.

Source of Variance	Sum of Squares	Degree of Freedom	Mean Square	*p*-Value
Model	70.18	12	5.85	<0.0001
*A*	0.53	1	0.53	<0.0001
*B*	1.26	1	1.26	<0.0001
*C*	38.17	1	38.17	<0.0001
*AB*	1.21 × 10^−4^	1	1.21 × 10^−4^	<0.0001
*AC*	9.41 × 10^−3^	1	9.41 × 10^−3^	<0.0001
*BC*	0.052	1	0.052	<0.0001
*A* ^2^	0.18	1	0.18	<0.0001
*B* ^2^	0.2	1	0.2	<0.0001
*C* ^2^	9.81 × 10^−3^	1	9.81 × 10^−3^	<0.0001
*A* ^2^ *B*	0.2	1	0.2	<0.0001
*A* ^2^ *C*	0.31	1	0.31	<0.0001
*AB* ^2^	6.48 × 10^−4^	1	6.48 × 10^−4^	<0.0001

**Table 8:** Analysis of variance for the regression equation of bending force *F*x.

Source of Variance	Sum of Squares	Degree of Freedom	Mean Square	*p*-Value
Model	62.93	12	5.24	<0.0001
*A*	0.014	1	0.014	<0.0001
*B*	4.1 × 10^−3^	1	4.1 × 10^−3^	<0.0001
*C*	28.41	1	28.41	<0.0001
*AB*	0.05	1	0.05	<0.0001
*AC*	0.086	1	0.086	<0.0001
*BC*	1.02 × 10^−3^	1	1.02 × 10^−3^	<0.0001
*A* ^2^	0.94	1	0.94	<0.0001
*B* ^2^	0.42	1	0.42	<0.0001
*C* ^2^	0.46	1	0.46	<0.0001
*A* ^2^ *B*	2.28 × 10^−3^	1	2.28 × 10^−3^	<0.0001
*A* ^2^ *C*	0.071	1	0.071	<0.0001
*AB* ^2^	2.96 × 10^−3^	1	2.96 × 10^−3^	<0.000 1

## Data Availability

The data that support the findings of this study are available from the corresponding author upon reasonable request.
